# Researching the Welfare Impact of Populist Radical Right Parties Comment on "A Scoping Review of Populist Radical Right Parties’ Influence on Welfare Policy and its Implications for Population Health in Europe"

**DOI:** 10.34172/ijhpm.2020.125

**Published:** 2020-07-18

**Authors:** Alexandru D. Moise

**Affiliations:** Department of Political and Social Sciences, European University Institute, Fiesole, Italy.

**Keywords:** Populist Radical Right, Health Policy, Welfare Chauvinism, Partisanship

## Abstract

Populist radical right (PRR) parties can impact population health through multiple mechanisms, including welfare chauvinistic policies, influencing mainstream parties, and eroding democratic norms. Rinaldi and Bekker survey the literature in order to motivate a wider research agenda. They highlight results from existing studies which show the importance of looking into the impact of PRR parties on welfare policy. This commentary considers some of the areas of research highlighted by the original article, as well as other possibilities for further research. The most important of these is to expand the sample of cases to Central and Eastern Europe, Latin America, and South East Asia.

## Introduction

Political parties shape the policies that ultimately impact the health and well-being of citizens. While a party’s behavior is influenced by their voters and interest groups, and mediated through political and welfare institutions, party ideology continues to be a driving force that can explain policy outcomes. Traditionally, researchers have focused on the distinction between left and right parties. The former was expected to increase welfare generosity, while the latter was expected to retrench it. However, the relevance of left-right as the main dimension that differentiates parties has long been questioned.^1^ More recently, Häusermann and Kriesi^2^ show the relevance of a cultural dimension, which can explain why electorates are changing. Their research finds that wealthier and more educated voters turn to left-wing parties, while working class voters have turned to conservative or populist parties.

The changing patterns of voter alignment, together with the rise of new party types, open a fresh set of questions regarding the role of parties and their ideology in shaping policy. The rise of populist radical right (PRR) parties is particularly important, as their impact on welfare policies is still uncertain. PRR ideology, focused on populism, nativism, and authoritarianism,^3^ does not seem to have direct implications for health policy, in the same way that it does for immigration or economic policy. Despite this, PRR parties still influence the welfare state in ways that seem to be distinct from centrist parties.

Rinaldi and Bekker^4^ are among the first to systematically explore the relationship between this party type and healthcare, proxied through the more general category of welfare policy. Using this broader category is understandable, given the scarcity of studies looking directly at health policy. Moreover, the intent of the article is to point out this gap in research and help generate momentum to fill it. By using a scoping review of peer-reviewed research, they identify 15 empirical papers that try to assess the impact of PPR on welfare policy. The studies all focus on PRR parties in Western European countries, most often in Austria, Switzerland, Denmark, Italy, and Sweden.

The common finding across papers is that PRR parties engage in welfare chauvinism, which presumes increasing welfare provisions for the native population while excluding minorities and immigrants.^5^ PRR parties across Western Europe promoted these policies while in governing coalitions with right-wing parties, but also while out of government, by putting pressure on both left and right parties.

 The second major finding of the review is that these policies are mediated through institutions. Since welfare chauvinism seeks to restricts rights of access to services, Rinaldi and Bekker point to studies that show how judicial systems, either national or European, have challenged PRR policies. Existing welfare institutions, such as whether the health system is tax-based or social-insurance based, also seem to influence the behavior of PRR parties. Rinaldi and Bekker point to the greater preponderance of welfare chauvinistic claims in tax-based system and speculate that they are easier targets as they are more at odds with PRR ideology.

 Partisanship can also act as a mediator for PRR policy since the parties need to make a choice between office-seeking behavior and vote-seeking behavior. The former refers to making compromises with center-right parties in order to join the governing coalition, while the latter supposes following their own radical agenda in order to satisfy their voters. Beyond acting as a mediator, looking at partisanship also shows us how the PRR can leverage influence over center-right and even center-left parties, by challenging their electoral base.

## Populist Radical Right Welfare and Healthcare

There are important reasons, moving forward, for why scholars should focus on distinct policy types when analyzing the importance of parties and the role of institutions in mediating their impact. One of the most important reasons as to why healthcare, together with pensions, should be treated separately, is that it is a life-course risk.^6^ Quite differently from unemployment and other labor-market risks, which affects working-class individuals more, healthcare is a universal risk, for which both the rich and poor need protection. This has profound implications for parties, given that this means that public healthcare has generally higher support which can cut across income groups, and voters perceive recipients of healthcare as more deserving than those of unemployment.^7^

The implications of these specificities of health policy for right-wing parties is that when it comes to health, they are more constrained given that their electorate is split on the issue.^8^ However, the implications for PRR parties still remain to be unpacked. It is an open question as to whether the special status of healthcare would make it easier or harder for PRR parties to focus on it with welfare chauvinist reforms. Given that healthcare, similarly to pensions, is more popular among older voters, and generally more popular with the electorate at large, it seems plausible that the PRR would seek to expand access at least for the native population. Liberal chauvinism should therefore be less likely in healthcare, as compared to other policies. The perception of greater deservingness of healthcare recipients should also make it more difficult for PRR parties to justify welfare chauvinism.

## Worlds of Welfare Chauvinism

Rinaldi and Bekker find that most PRR parties in Western Europe engaged or attempted to engage in some form of welfare chauvinism. They discuss that welfare chauvinism is a natural extension of the PRR ideology, particularly of nativism and authoritarianism. However, not all PRR parties share the same ideological tenants, and not all of them focus on healthcare. Moving forward, scholars should seek explanations as to *why* PRR parties might focus on healthcare and on a particular type of chauvinism. The answer likely lies in their distinct electorates. While working-class electorates have partly shifted to PRR parties, the exact patterns are likely to differ across countries. PRR parties in Central and Eastern Europe have less of a focus on immigration than their Western counterparts. They also seem more successful in diversifying their voter base beyond the working class. The preferences of their different voter groups will likely explain much of the variation in PRR parties’ behavior.

Part of this variation refers to different types of approaches to welfare. Besides welfare chauvinism, scholars have argued that PRR parties also engage in welfare populism^9^ and liberal welfare chauvinism.^10^ Welfare populism combines economic egalitarianism with a critique of the welfare state as not serving the “common man.” Parties following this pattern attempt to expand access or benefits for poorer individuals (of the native population), while criticizing or attacking bureaucratic structures perceived to be elitist or corrupt. Liberal chauvinism combines the nativist restrictions of services with a more conservative view of reducing all welfare provisions. Examples of this type are the Austrian Freedom Party, and the Italian Northern League. As Rinaldi and Bekker highlight, the choice of parties between these and other approaches will depend on the degree to which they wish to satisfy their voters and their coalition partners.

## Mediating the Impact of Populist Radical Right Ideology

Rinaldi and Bekker discuss a number of mediating factors affecting the ability of PRR parties to implement policies. While the studies they review suggest that tax-based systems are more vulnerable to welfare chauvinism, more research is needed to clarify this. Existing research tells us that tax-based universal systems are the hardest to reform due to institutional path dependence as well as the popularity of universal programs among voters.^11^ Even if these systems are likelier targets of PRR rhetoric, existing research tells us that there is a trade-off between this rhetoric and the actual ability of parties to pass restrictive policies. If further research does indeed show that PRR parties have an easier time reforming tax-based systems, this would make them more successful than conservative parties, and thus an even more interesting case to study.

More research is also needed to understand the role of judicial institutions in mediating policy. Traditionally, the courts have been used as a veto-point by opposition parties or interest groups to block reforms.^12^ For example, the Czech Social Democrats, effectively challenged copayments introduced in the health system, which led to their eventual repeal.^13^ Given that welfare chauvinism seeks to limit the rights of immigrants or minorities, courts might prove to be even more important for opposition parties to block such reforms.

 Further research should also incorporate political institutions more broadly. Other veto points, including the number of chambers in the legislature, presidential veto, and referenda, as well as other institutions such as electoral systems, likely play a role not just in blocking reforms, but more generally in shaping the behavior and electoral strategies of PRR parties as they try to gain more votes and pass policy.

## Broadening the Sample

 The final important step for researchers moving forward is to broaden the sample of PRR parties to Central and Easter Europe, and then further to Latin America, South East Asia, and other cases. Broadening the sample can answer important questions that are inaccessible when looking only at Western Europe. One question is what PRR parties do, once they are the main party in government. The dilemma that the review highlights, between office-seeking and vote-seeking behavior, is specific to the smaller PRR parties in Western Europe, which need to rely on center-right parties as a means of entering government. In Poland and Hungary, however, the trade-off does not exist in this way, since office-seeking and vote-seeking represent the same strategy, as the PRR parties are large enough to form single-party governments.

Poland and Hungary also offer a glimpse into the different policies that PRR parties might pursue once they control government. In Poland, the PiS government, while following some degree of welfare chauvinism, also proposed (and later abandoned) a reform meant to move the health system to a tax-based one.^14^ In Hungary, Fidesz, while in opposition, opposed copayments and other marketization reforms. Once in power, they renationalized hospitals.^15^ Further research should clarify to what degree such reforms are examples of welfare chauvinism, liberal chauvinism, welfare populism, or if they are a distinct policy strategy.

 These are only some of the questions that are not answerable by focusing solely on Western European parties. Researchers are bound to uncover other questions, as well as additional theoretical insights once other cases are carefully analyzed.

## Possibilities for a New Research Agenda on PRR Parties

 PRR parties have been gaining considerable ground over the last decade, and it seems implausible that they will disappear any time soon. It is therefore important to ask how these parties impact the health of citizens. As Rinaldi and Bekker show in their review, and as this commentary re-emphasizes, there are likely multiple mechanisms through which these parties impact population health. By restricting the access of vulnerable groups to healthcare, PRR policies damage their health. The impact of these parties on the rule of law and on the policy positions of mainstream parties are more indirect mechanisms through which health is affected.

 It is therefore more important than ever to look at the impact that these parties have on public health. Important questions remain to be answered by scholars. Why is it that some PRR parties emphasize healthcare in their manifestos and in their coalition negotiations, while other only focus on immigration and security? Why are these parties successful in some countries and not in others, and is this linked to their stances on healthcare? How do PRR parties behave once they obtain more power and can form a government? How do political institutions shape their policy decisions and preferences? And lastly, what does the composition of the PRR electorate in different countries mean for the vote-seeking behavior of these parties? Rinaldi and Bekker’s review effectively raises some of these questions, and in doing so does a service to the community of scholars tackling these issues.

## Ethical issues

 Not applicable.

## Competing interests

 Author declares that he has no competing interests.

## Author’s contribution

 ADM is the single author of the paper.
